# New insights into the type A glycan modification of *Clostridioides difficile* flagellar protein flagellin C by phosphoproteomics analysis

**DOI:** 10.1016/j.jbc.2022.101622

**Published:** 2022-01-20

**Authors:** Paul J. Hensbergen, Arnoud H. de Ru, Annemieke H. Friggen, Jeroen Corver, Wiep Klaas Smits, Peter A. van Veelen

**Affiliations:** 1 Center for Proteomics and Metabolomics, Leiden University Medical Center, Leiden, The Netherlands; 2 Department of Medical Microbiology, Leiden University Medical Center, Leiden, The Netherlands

**Keywords:** phosphonate, motility, *Clostridioides*, mass spectrometry, IMAC, glycosylation, 2-aminoethylphosphonate, 2-AEP, 2-aminoethylphosphonate, CDI, *Clostridioides difficile* infection, EThcD, electron-transfer/higher-energy collision dissociation, FA, formic acid, FliC, flagellin C, HCD, high-energy collision dissociation, IMAC, immobilized metal affinity chromatography, pGlcNAc, phosphorylated GlcNAc, PTM, post-translational modification

## Abstract

The type A glycan modification found in human pathogen *Clostridioides difficile* consists of a monosaccharide (GlcNAc) that is linked to an *N*-methylated threonine through a phosphodiester bond. This structure has previously been described on the flagellar protein flagellin C of several *C. difficile* strains and is important for bacterial motility. The study of post-translational modifications often relies on some type of enrichment strategy; however, a procedure for enrichment of this modification has not yet been demonstrated. In this study, we show that an approach that is commonly used in phosphoproteomics, Fe^3+^-immobilized metal affinity chromatography, also enriches for peptides with this unique post-translational modification. Using LC–MS/MS analyses of immobilized metal affinity chromatography–captured tryptic peptides, we observed not only type A-modified *C. difficile* flagellin peptides but also a variety of truncated/modified type A structures on these peptides. Using an elaborate set of mass spectrometry analyses, we demonstrate that one of these modifications consists of a type A structure containing a phosphonate (2-aminoethylphosphonate), a modification that is rarely observed and has hitherto not been described in *C. difficile*. In conclusion, we show that a common enrichment strategy results in reliable identification of peptides carrying a type A glycan modification, and that the results obtained can be used to advance models about its biosynthesis.

In addition to the wealth of post-translational modifications (PTMs) encountered in the proteome of eukaryotic cells, it is well known that the microbial world presents us with an even higher variety of post-translationally modified structures (1). Their identification has shown us how remarkable such modifications can be, and the underlying biosynthetic pathways also revealed enzymes with unprecedented activities (2, 3, 4, 5). Moreover, the characterization of the modifications, both structurally and functionally, is important to understand pathogenic mechanisms of infection and virulence.

For the study of PTMs, a wide variety of different approaches is used. Some of these approaches focus on individual proteins, for example, by the use of protein-specific phosphoantibodies to study cell signaling events. More global analyses of PTMs often depend on purification steps to enrich for modified proteins/peptides. For example, in glycoproteomics, lectin affinity approaches (6, 7) and hydrophilic interaction chromatography (8) have been widely used. In phosphoproteomics, the most common techniques are immobilized metal affinity chromatography (IMAC) and titanium dioxide affinity purification (9). Even though they are primarily used for the enrichment of simple phosphorylated peptides, it has recently been shown that such an approach can also be useful for the study of mannose-6-phosphate-modified peptides (10).

*Clostridioides difficile* infection (CDI) is a well-known cause of health care–associated diarrhea, usually associated with antibiotics usage (11). *C. difficile* is an anaerobic gram-positive spore-forming bacterium that can reside within the commensal microbiome. The symptoms of CDI are primarily caused by the activity of toxins (12). In severe cases, CDI can develop into, for instance, pseudomembranous colitis, which can be lethal. Because *C. difficile* spores can survive for months on contaminated surfaces and tools, it is a problem for health care institutes, especially when outbreaks of virulent strains occur.

For an efficient infection, the balance between adhesion and motility is essential. The motility of *C. difficile* largely depends on the presence of flagella, which are complex cell surface structures composed of many different protein subunits. In addition to motility, flagella also contribute to adherence, which is an important prerequisite for colonization (13, 14, 15). The filament of flagella is composed of polymeric structures of flagellin C (FliC). Of note, a *fliC* knockout strain of *C. difficile* showed increased levels of toxin production (16, 17), demonstrating a close association between processes involved in motility and colonization, and other major virulence factors. In *C. difficile*, FliC is modified with unusual glycan modifications (18, 19). One of these (type A, Fig. 1), which is also present in the widely used laboratory strain 630Δ*erm*, consists of an *O*-GlcNAc (to Ser and Thr residues), linked, through a phosphodiester bond, to an *N*-methyl-l-threonine (19). The absence of this modification impairs motility.

**Figure 1:**
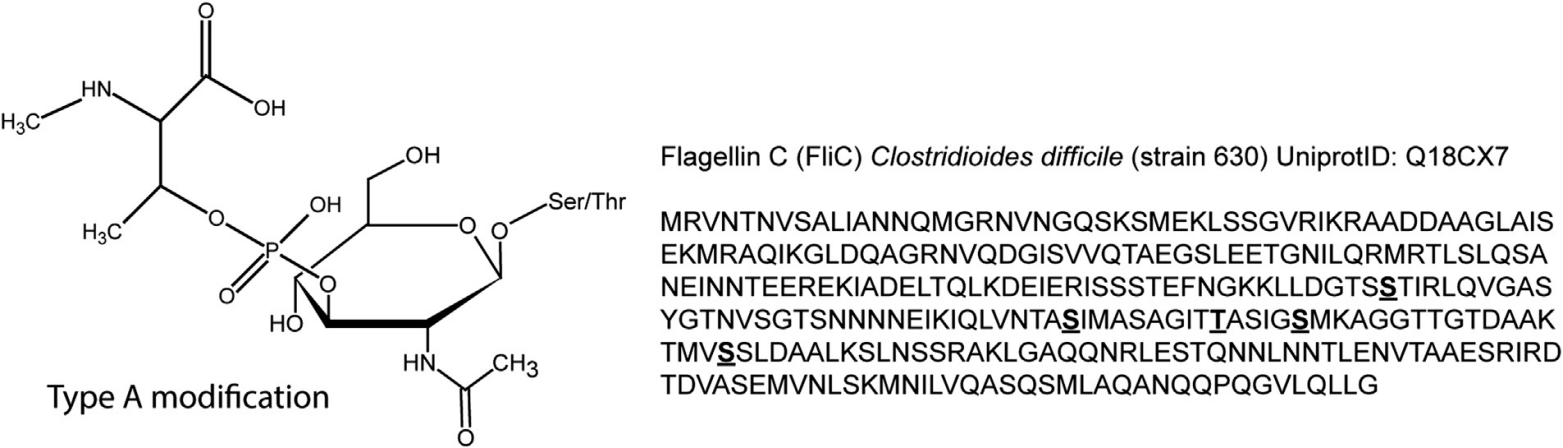
**Structure of the type A modification of *Clostridioides difficile* strain 630Δ*****erm*****flagellin C (FliC).***Left*, structure of the type A modification consisting of an *O*-linked GlcNAc, linked, through a phosphodiester, to an *N*-methyl-l-threonine. *Right*, type A-modified serine and threonine residues of *C. difficile* strain 630 FliC (UniProt ID: Q18CX7), *bold* and *underlined*. Based on Faulds-Pain *et al.* (19) and Twine *et al.* (15).

In this article, we show that Fe^3+^-IMAC also enriches for type A-modified peptides from a complete trypsin-digested *C. difficile* proteome. Such peptides can be clearly recognized by specific low-mass fragments, which allowed us to also recognize variations in the type A structure that were hitherto unknown. Through detailed tandem mass spectrometry (MS/MS) experiments, we show that one of these corresponds to the addition of an unusual phosphonate moiety, 2-aminoethylphosphonate (2-AEP).

## Results

### Fe^3+^-IMAC is effective for the enrichment of atypical phosphomodified peptides (type A peptides) from *C. difficile*

**Figure 2:**
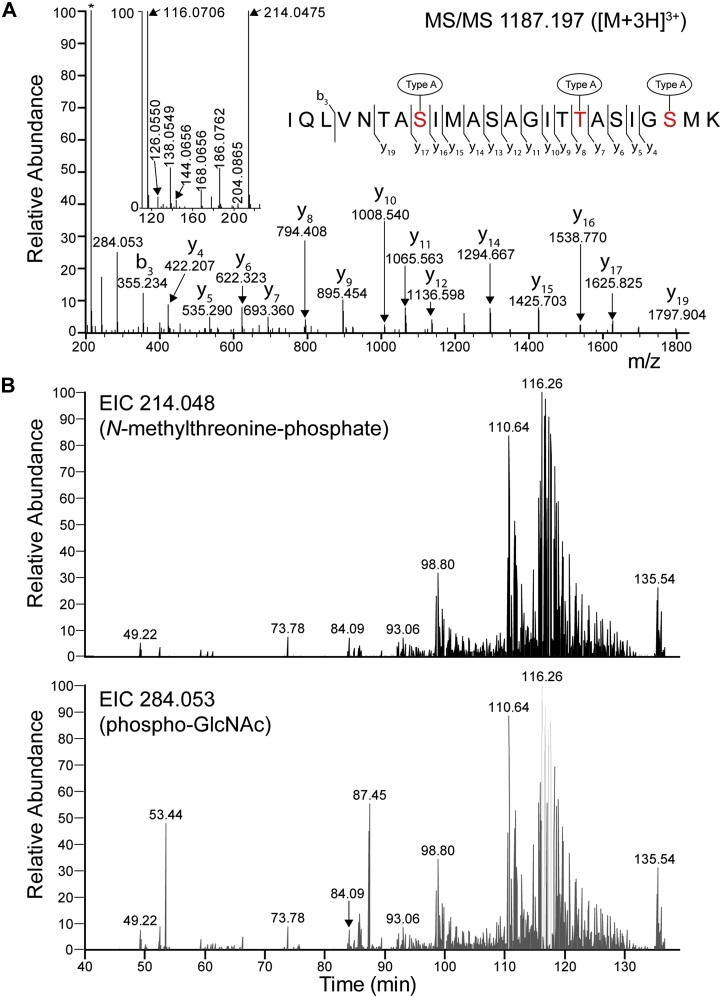
**Identification of type A-modified flagellin C (FliC) peptides from *Clostridioides difficile* strain 630Δ*****erm*****after Fe**^**3+**^**-IMAC enrichment.***A*, a total tryptic digest of a protein extract of *C. difficile* was processed using Fe^3+^-IMAC, and bound peptides were collected after high-pH elution and analyzed by LC–MS/MS analysis. The MS/MS spectrum of the most abundant peptide (*m/z* 1187.197, [M + 3H]^3+^) corresponds to the tryptic peptide IQLVNTASIMASAGITTASIGSMK of FliC, carrying three type A structures. In addition to the ions at *m/z* 214.048 (*N*-methylthreonine-phosphate, indicated with an *asterisk* [∗]) and 284.053 (phospho-GlcNAc), this spectrum is characterized not only by several GlcNAc oxonium ions (*i.e.*, at *m/z* 204.0865, 186.0762, 168.0656, 144.0656, and 138.0549) but also by a fragment at *m/z* 116.0706 (*N*-methylthreonine residue mass) (see *inset*). The series of b ions and y ions was all unmodified as a result of the neutral loss of the type A modification. EThcD fragmentation (Fig. S2) confirmed the presence of the type A modification on Ser-8, Thr-17, and Ser-22 of this peptide. *B*, the general presence of type A-modified peptides in the IMAC-bound fraction was evident by the presence of the characteristic fragment ions at *m/z* 214.048 (*N*-methylthreonine-phosphate, *upper panel*) and *m/z* 284.053 (phospho-GlcNAc, *lower panel*), in the MS/MS data, as shown by their respective extracted ion chromatograms (EICs). EThcD, electron-transfer/higher-energy collision dissociation; IMAC, immobilized metal affinity chromatography.

In data from our ongoing studies of protein phosphorylation in *C. difficile* (20), where we combine Fe^3+^-IMAC of tryptic peptides with high-accuracy LC–MS/MS analyses, we observed that the major signals in our chromatogram were not explained by standard S/T/Y phosphopeptides. The MS/MS spectrum of the major ion species (at *m/z* 1187.197, Fig. 2*A*) was manually inspected. Based on the series of ions, a sequence tag was used for a BLAST search, which resulted in a hit with *C. difficile* FliC. We also noticed some characteristic HexNAc oxonium ions in the low-mass region of the spectrum (*e.g.*, at *m/z* 204.087, 186.076, 168.066, 144.066, 138.055, and 126.055). Moreover, this region was dominated by ions that were previously found to be specific for the type A glycan ((15), Fig. 1). The specific ions were observed at *m/z* 214.048 (*N*-methylthreonine-phosphate, [M + H]^+^) and *m/z* 284.053 (phospho-GlcNAc, [C_8_H_15_NO_8_P]^+^). In general, these ions were frequently observed in the MS/MS data as evident from extracted ion chromatograms of these ions in our data (Fig. 2*B*). Furthermore, the presence of these ions in the fragmentation spectra showed a high degree of correlation (Fig. 2*B*, *upper* and *lower panels*), demonstrating that they are derived from the same precursor ions. The pattern of these ions was very similar in the analyses of three biological culture replicates that were independently processed by Fe^3+^-IMAC (Fig. S1).

Overall, we found that the major peptide that we observed in our IMAC-purified material corresponds to the tryptic FliC peptide, IQLVNTASIMASAGITTASIGSMK, carrying three type A modifications (Fig. 2*A*). In addition to a series of (unmodified) b ions and y ions, and the low-mass ions described previously, the MS/MS spectrum of this peptide showed an ion at *m/z* 116.071 (Fig. 2*A*, *inset*), corresponding to the residue mass of *N*-methylthreonine ([M + H]^+^). We also performed electron-transfer/higher-energy collision dissociation (EThcD) fragmentation (combining electron transfer dissociation with high-energy collision dissociation [HCD], (21)) of this peptide, which showed that Ser-8, Thr-17, and Ser-22 are modified (Fig. S2, residues in *red* in Fig. 2*A*).

### *C. difficile* FliC is modified with a variety of type A (sub)structures

As can be observed in Figure 2*B*, some MS/MS spectra contain the ion at *m/z* 284.053 but not the ion at *m/z* 214.047. Close inspection of these MS/MS spectra showed that they correspond to peptides modified with a single phosphorylated GlcNAc (pGlcNAc). For example, the signal at 87.45 min (Fig. 2*B*, *lower panel*) corresponds to the tryptic peptide TMVSSLDAALK with a pGlcNAc (*m/z* 709.828, [M + 2H]^2+^, Fig. S3*A*). In addition to the absence of the ion at *m/z* 214.048, this spectrum also lacks the ion at *m/z* 116.071 described previously, supporting the structural assignment. In our data, the overall intensity of this peptide is lower than the corresponding peptide with the full type A structure (*m/z* 767.360 ([M + 2H]^2+^, Fig. S3*B*). These data exemplify the presence of truncations of the type A structure on FliC in our data. To rigorously study the extent of these substructures, we performed a database search that identified 619 peptide spectrum matches from flagellin peptides modified with a type A (sub)structure (Table S1) in a single run. In total, six different FliC tryptic peptides had at least one type A structure, covering the region of amino acids 133 to 212 (Fig. 1). As expected, the highest number of peptide spectrum matches (82%) was annotated to the tryptic peptide IQLVNTASIMASAGITTASIGSMK, on which a high diversity of modifications was found. Most of these corresponded to the peptide indicated in Figure 2*A* (with three canonical type A structures), but many different variations thereof were observed (Table S1). For example, peptides were modified with a combination of type A structures without the methyl group, pGlcNAc, or GlcNAc (Table S1).

### Part of the type A structures carries an extra methyl group

**Figure 3:**
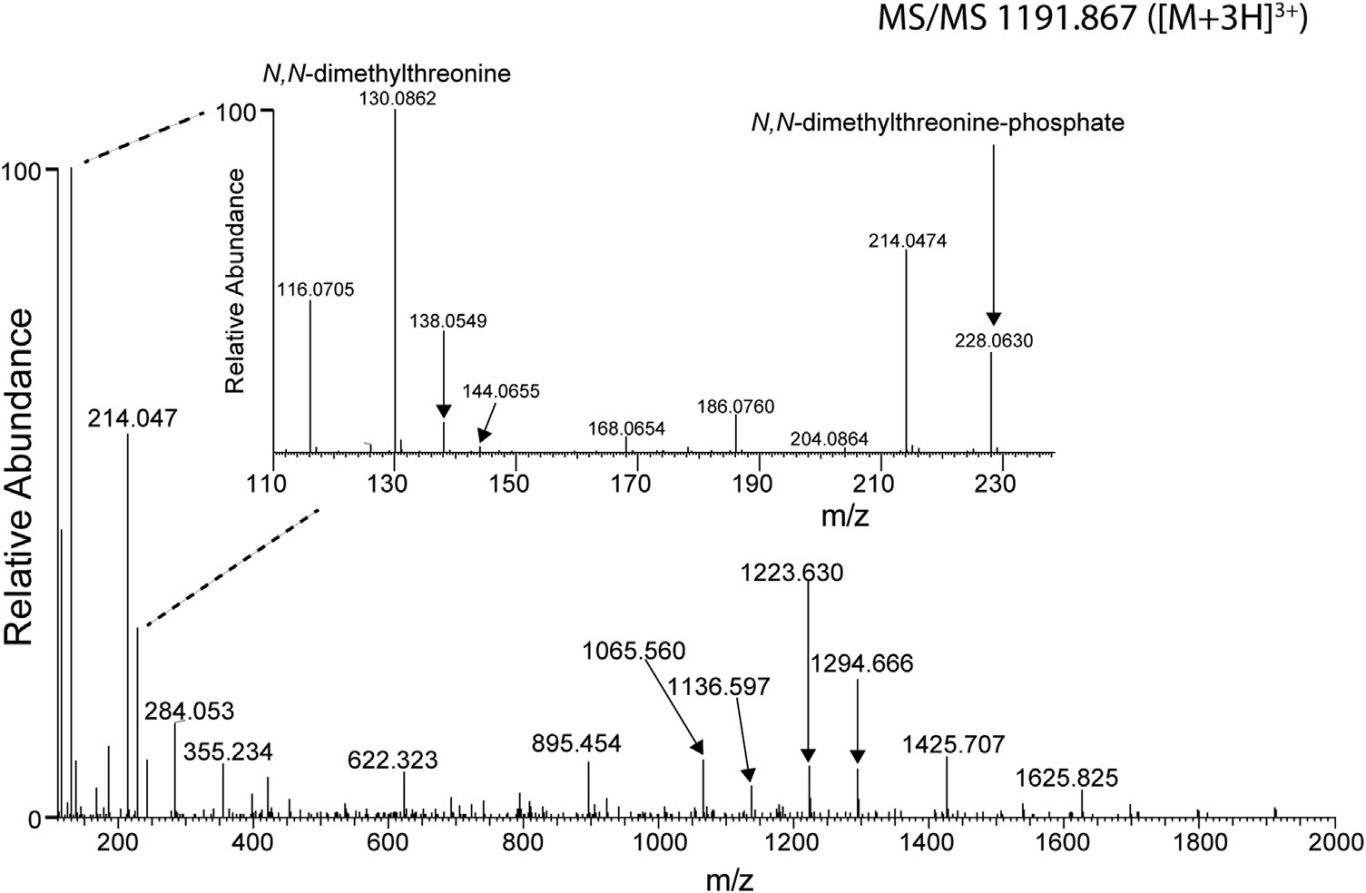
**Identification of a novel type A structure with an extra methyl group.** MS/MS spectrum of the flagellin C tryptic peptide IQLVNTASIMASAGITTASIGSMK carrying three type A structures (Fig. 1*A*) in which one has an extra methyl moiety (*m/z* 1191.867 [M + 3H]^3+^). This MS/MS spectrum is characterized by the specific fragment ion at *m/z* 130.0862, which is tentatively assigned as a *N*,*N*-dimethylthreonine. The corresponding phosphate ion was observed at *m/z* 228.0630.

Further examination of our data for other possible variations of the type A structure suggested that some structures have additional methylation. For example, next to the major modified peptide described previously (*m/z* 1187.176, [M + 3H]^3+^), IQLVNTASIMASAGITTASIGSMK + three type A structures), we observed an ion with an additional mass corresponding to a methyl group (*m/z* 1191.867 [M + 3H]^3+^). The MS/MS spectrum of this peptide (Fig. 3) was indeed similar to that in Figure 2*A*, but in addition to the ion at *m/z* 116.071 (*N*-methylthreonine), an intense ion at *m/z* 130.086 was also apparent. This matches with the mass of an *N,N*-dimethylthreonine (theoretical *m/z* 130.086). In line with this, the ion at *m/z* 228.063 forms a corresponding doublet with the ion at *m/z* 214.047. The presence of the ions at *m/z* 116.071 and 214.047 in this spectrum can be explained by the fact that this peptide also contains two canonical type A structures, in addition to the one with the extra methyl group.

### The GlcNAc of type A can be modified with a phosphonate: 2-AEP

**Figure 4:**
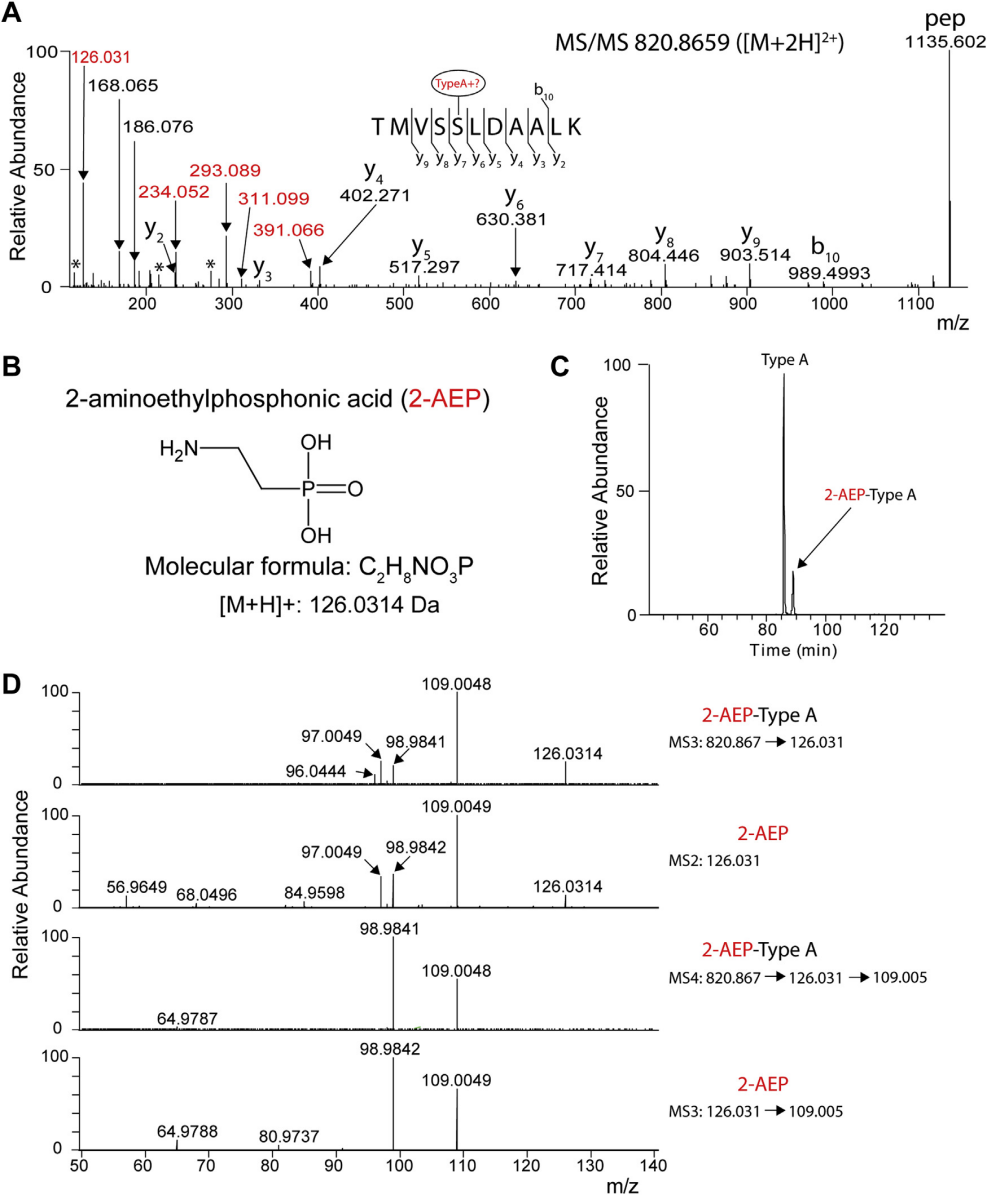
**The type A structure can be modified with 2-aminoethylphosphonate (2-AEP).***A*, MS/MS spectrum of the flagellin C tryptic peptide TMVSSLDAALK, modified with an alternative type A structure, indicated in *red* as “type A+?”). In addition to the type A characteristic fragments indicated in Figure 2, this spectrum is characterized by several other fragment ions (*e.g.*, at *m/z* 126.031, 234.052, 293.089, 311.0994, and 391.066, labeled *red*). The b ions and y ions lost the modification. The site assignment is based on Figure 1, based on the accurate mass, the ion at *m/z* 126.0313 (see *A*) was identified as the natural product 2-aminoethylphosphonic acid. Phosphonates are characterized by a carbon–phosphorous bond. *C*, chromatographic profile of the tryptic peptide TMVSSLDAALK modified with either a type A or 2-AEP type A structure. *D*, comparison of the fragmentation pattern of synthetic 2-AEP and a 2-AEP-type A-modified peptide. The *upper two panels* show the MS3 spectrum of the peptide TMVSSLDAALK modified with a 2-AEP type A structure and the MS2 spectrum of synthetic 2-AEP. The *lower two panels* show additional fragmentation of the major ion (at *m/z* 109.005) observed in the *upper two panels*.

As mentioned previously, the fragmentation spectra from the type A-modified peptides are characterized by specific low-mass fragments such as not only the ions at *m/z* 214.048 and 284.053 but also the commonly observed oxonium ions from the GlcNAc. In our data, we also found several fragmentation spectra from putative type A-modified tryptic FliC peptides where additional intense fragment ions at, for example, *m/z* 126.031, 234.052, 293.089, 311.099, and 391.066, were observed. For example, Figure 4*A* shows such an MS/MS spectrum from peptide TMVSSLDAALK, which appeared to be modified with a modified type A structure (indicated in *red*). The mass of this peptide (*m/z* 820.8659 ([M + 2H]^2+^) differs from that of a typical type A-modified peptide (*m/z* 767.3594 ([M + 2H]^2+^, Fig. S4) by an additional mass of 107.013 Da. Interestingly, this could explain the fragment that was observed at *m/z* 126.031 (107.013 + H_2_O + H^+^, Fig. 4*A*). Given the fact that the type A-specific fragments at *m/z* 116.071, 214.048, and 284.053 are still present in the MS/MS spectrum (even though at lower relative intensities, indicated with an ∗ in Fig. 4*A*), it is most likely that the additional mass is not present on the *N*-methylthreonine-phospho moiety but at another position on the GlcNAc core. This is supported by the fragment at *m/z* 311.099 (GlcNAc oxonium ion at 204.086 + 107.013).

To identify the unknown moiety, we used the accurate mass of the ion at *m/z* 126.031 for the prediction of the elemental composition. Using a tolerance of 5 ppm (which was appropriate given the accuracy that we observed for the low-mass HexNAc oxonium ions mentioned previously), this resulted in two possibilities: C_6_H_6_O_3_ and C_2_H_9_NO_3_P. Assuming the *m/z* 126.031 to be a protonated species, the nitrogen rule dictates the presence of an odd number of nitrogen atoms for the odd nominal neutral mass (*i.e.*, 125), leaving C_2_H_9_NO_3_P as the only possible molecular formula, even though this option also struck us as unusual because it contains phosphorus but only three oxygens and would therefore not be compatible with a phosphate. However, a PubChem search with C_2_H_8_NO_3_P led to a candidate natural product, 2-aminoethylphosphonic acid (Fig. 4*B*). Importantly, this moiety contains a carbon–phosphorous bond (*i.e.*, it is a phosphonate), something that is not commonly observed in nature. In our IMAC-enriched peptide pool, a small, but significant, amount of the type A appeared to be modified with the 2-AEP moiety (Fig. 4*C*).

To corroborate the assignment of the extra moiety on the type A structure as 2-AEP, we performed additional fragmentation experiments of the ion at *m/z* 126.031 and compared it with the MS/MS spectrum of synthetic 2-AEP. More specifically, we set up targeted MS/MS analyses and compared the MS3 spectrum from the modified peptide TMVSSLDAALK (selecting for the second round of fragmentation the species at *m/z* 126.031) and compared that with the MS2 spectrum of synthetic 2-AEP. As shown in Figure 4*D* (first and second panels), these two fragmentation spectra are highly similar. The most abundant ion in these spectra was found at *m/z* 109.005. Subsequent fragmentation of this species also resulted in highly similar spectra in both cases (Fig. 4*D*, *third* and *fourth panels*). Of note, experiments with the isomeric molecule 1-AEP showed that its fragmentation rigorously differed from that of 2-AEP (results not shown).

To provide more mechanistic insight into the fragmentation patterns observed in Figure 4*A*, and support our hypothesis that the 2-AEP moiety is on the GlcNAc, we also performed additional fragmentation experiments of the ions at *m/z* 293.089 and 391.066 (Fig. 4*A*), which have a mass difference corresponding to a phosphate.

Importantly, fragmentation of both species resulted in the 2-AEP ion at *m/z* 126.031 (Fig. S5) and the common GlcNAc oxonium ions mentioned previously. Another fragment ion was observed at *m/z* 204.042, which is evidently different from the GlcNAc oxonium ion at *m/z* 204.086. The ion at *m/z* 204.042 perfectly matches with an oxonium ion where all groups (including the *N*-methylthreonine-phosphate moiety) are lost from the GlcNAc ring, except for the 2-AEP moiety. This ion forms a doublet with the ion at *m/z* 234.052 (+CH_2_O). These ions provide further support for the fact that the 2-AEP moiety is indeed present on the GlcNAc. An overview of the observed 2-AEP-containing fragments and their proposed elemental composition and structures can be found in Fig S5*B*.

Overall, the aforementioned data provide strong evidence for the presence of 2-AEP on the type A modification of *C. difficile* FliC. Based on our in-depth MS fragmentation experiments, we propose that the 2-AEP moiety is attached to the C4 position of the GlcNAc.

## Discussion

The study of PTMs of peptides and proteins by MS is generally dependent on enrichment methods. In this article, we demonstrate that a common approach used in phosphoproteomics (Fe^3+^-IMAC) also enriches for peptides that are modified with a unique *C. difficile* FliC glycan modification (type A). Based on our current experiments, we do not know whether the binding can solely be explained by the presence of the phosphomoiety or that other structural determinants also play a role. Moreover, using this method in combination with extensive fragmentation experiments, we identified a broad range of variations of the type A structure. Most of these were truncations of the type A structure, but others represented additional modifications, that is, an extra methyl group and a phosphonate, 2-AEP.

We found a set of type A-modified tryptic FliC peptides, which confirms that the region between amino acids 133 and 212 of FliC is modified with this structure. In earlier experiments, analyses of the type A modification were performed using high amounts of purified FliC in combination with manual inspection of the fragmentation spectra (19). Our analysis, where we used a total tryptic digest of *C. difficile* proteins, indeed showed that FliC is the most important, if not the only, protein modified with the type A structure in *C. difficile* because on the basis of our database search in combination with manual inspection, no other hits were found.

Our data were dominated by a tryptic FliC peptide on which three type A structures were found, which may reflect the high affinity of this peptide for the Fe^3+^-IMAC material. In previous experiments, a maximum of seven type A modification sites in FliC were found (15). The results from our database search provide evidence for more possible sites that can be modified with the type A glycan structure, but as a result of our bottom–up approach, we cannot determine the number per intact FliC molecule.

HCD MS/MS spectra of type A-modified peptides are dominated by characteristic low-mass ions, which aid in their assignment and identification in complex datasets, especially on high-resolution MS instruments. Originally, the type A structure was fully elucidated through NMR analysis, which showed that the HexNAc is a GlcNAc, and that the *N*-methylthreonine-phospho moiety is attached to the C3 position (19). It has recently been suggested that the ratio between the abundance of the HexNAc oxonium ions at *m/z* 138.055 and 144.065 in MS/MS spectra of *O*-glycosylated peptides can be used to discriminate between a GlcNAc and GalNAc (22, 23). More specifically, a ratio >2 would be indicative of a GlcNAc, which is supported by the MS/MS spectra of type A-modified peptides presented in this article.

The observation of a variety of truncations of the type A structure may represent biosynthetic intermediates, and therefore, the assessment of such variations could provide insight into the different steps of the type A biosynthetic pathway. In *C. difficile* strain 630, a cluster of genes (*cd0240*–*cd0244*) is responsible for the synthesis of type A (15, 19). The first step is the addition of the GlcNAc to serine and threonine residues by the glycosyltransferase encoded by *cd0240*. The other four genes in the cluster (*cd0241*–*cd0244*) are involved in the subsequent addition of the *N*-methylthreonine-phospho moiety. One of the encoding proteins, CD0243, is a putative methyltransferase. Indeed, in a study where the alterations of the type A structure were studied in *C. difficile* mutant strains, lacking one of the genes in the cluster (19), peptides with a type A structure lacking the methyl moiety were observed in the *cd0243* knockout strain. Also in our data, this variation of the type A modification was present, probably as a result of incomplete type A biosynthesis. A definite function for the other three genes in the cluster (*cd0241*, *cd0242*, and *cd0244*) is hitherto not described, although based on the results previously published (19), a model was proposed where the threonine-phospho moiety is added in a single step, followed by methylation. Interestingly, this would not be compatible with the observation of phosphoHexNAc structures on the FliC peptides, as observed in our data. However, alternative explanations for their presence are possible, such as unknown enzymatic activity that can release the *N*-methylthreonine. Hence, further experiments are necessary to dissect the different steps involved in the biosynthesis of the type A modification, and the role of the different enzymes involved, and an IMAC-based method could be a valuable tool for such studies.

In the Gram-negative bacterium *Pseudomonas aeruginosa* PAO1 strains, a similar cluster of genes as found in the *C. difficile* strain 630 (and 630Δ*erm*) has been described. In these *P. aeruginosa* strains, the flagellin modification consists of an *O*-linked monosaccharide (in this case, a deoxyhexose), linked to an unknown moiety *via* a phosphate group. Hence, the flagellin modification in the PAO1 strain of *P. aeruginosa* has some similarity to the type A modification in *C. difficile*, but the full identity of the PAO1 modification remains elusive. Initial experiments indicated that the unknown moiety consists of a tyrosine residue (24), but subsequent studies by MS (25) determined a mass of 129.09 for this moiety. In line with this, a fragment at *m/z* 130.1 was observed in the MS/MS data. Interestingly, given the similarity of the gene clusters, including the presence of a CD0243 homolog in *P. aeruginosa* (PA1088), it is tempting to speculate that the unknown moiety is an *N,N*-dimethylthreonine, corresponding to one of the variations of the type A structure that we observed. Of note, we cannot formally rule out the possibility that the structure we have identified corresponds to the isomeric structure *N*-ethylthreonine, which would then imply that CD0243 also has ethyltransferase activity, but we deem this option less likely.

The most striking variation of the canonical type A structure that we describe in this study is the addition of the phosphonate, 2-AEP. In comparison to the more common phosphates, that contain carbon–oxygen bonds, phosphonates are characterized by a highly stable carbon–phosphorus bond. In some organisms, phosphonates appear to be the major inorganic phosphorous compound, such as in protozoan ciliates, where 2-AEP (alternative name ciliatine) was first identified (26). Even though phosphonates are hitherto not commonly described as part of larger biomolecules, phosphonolipids and phosphonoglycans have been identified (27, 28). For example, 2-AEP-modified glycans were found in snails (29), algae (30), jellyfish (31), and insects (32). We here provide evidence for 2-AEP modification of a GlcNAc as part of the type A modification of *C. difficile* flagellin. Our identification of the 2-AEP moiety was based on a set of detailed MS/MS experiments of 2-AEP type A-modified FliC tryptic peptides, in comparison with synthetic 2-AEP. Intriguingly, in these experiments, it was found that fragmentation of the 2-AEP ion at *m/z* 126.031 (and its fragment at *m/z* 109.005) resulted in a major species at *m/z* 98.984. This represents phosphoric acid, which can only be explained by a reaction with residual water in the collision cell, a phenomenon that has been described before (33).

To the best of our knowledge, 2-AEP-modified biomolecules have hitherto not been identified in *C. difficile*, and this raises the question about the origin of the 2-AEP. In all but one case, biosynthesis of phosphonates is dependent on PepM-mediated isomerization of phosphoenolpyruvate to phosphonopyruvate, usually followed by a decarboxylation reaction mediated by phosphonopyruvate decarboxylase. In a large-scale bioinformatics analysis looking for *pepM* homologs in microbial genomes, *C. difficile* came out as negative (34). On the contrary, the *C. difficile* genome encodes for enzymes as part of the carbon–phosphorus lyase system, which can use phosphonates as a source of phosphorous in case of phosphate starvation (35, 36). Hence, it remains to be established whether *C. difficile* is able to synthesize phosphonates through an alternative pathway, or that the bacterium takes it up from its environment. In the latter scenario, it is tempting to speculate that other bacteria may be a source of AEP *in vivo*. For example, several *Bacteriodes* species that are commonly found in normal gut microbiota have a PepM homolog (34), and among these, *Bacteriodes fragilis* is able to synthesize 2-AEP-modified capsular polysaccharide (37).

In conclusion, by scrutinizing data from Fe^3+^-IMAC enriched peptides, we have provided new insights in the variety of type A-modified peptides in *C. difficile*. The unique low-mass fragments of such peptides can be used for their assignment, and variations in these patterns allowed us to identify hitherto unknown variations in this structure, including the presence of an unusual phosphonate moiety (2-AEP). Our study provides a valuable approach to unravel the type A biosynthetic pathway not only in *C. difficile* but also in species with a similar modification, such as *P. aeruginosa*. Moreover, it opens up a route to the study of larger phosphonate-containing biomolecules.

## Experimental procedures

### Chemicals

General chemicals, aminoethylphosphonates (1 and 2-AEP), and complete mini EDTA-free protease inhibitors were purchased from Sigma–Aldrich.

### Cell culture and sample preparation

*C. difficile* 630Δ*erm* cells (38) were grown at 37 °C in brain–heart infusion medium supplemented with yeast extract (Thermo Fisher Scientific) in an anaerobic cabinet. Cell pellets from 50 ml overnight cultures were resuspended in 3 ml 8 M urea/50 mM Tris–HCl (pH 7.4), 1 mM orthovanadate, 5 mM Tris(2-carboxyethyl)phosphine, 30 mM chloroacetamide, phosphoSTOP phosphatase inhibitors (Thermo Fisher Scientific), complete mini EDTA-free protease inhibitors, and 1 mM MgCl_2_ and incubated for 20 min at room temperature. Cells were subsequently lysed by sonication (Soniprep 150 ultrasonic disintegrator; MSE), five times for 30 s at an amplitude of 12 microns. In between sonication steps, samples were cooled on ice for 30 s. Next, samples were centrifuged for 15 min at 7200*g* at 4 °C. The urea concentration was adjusted to 6 M by adding 1 ml of 50 mM Tris–HCl (pH 7.4) after which 1 μl benzonase (250 U/μl; Sigma–Aldrich) was added and samples were incubated for 2 h at room temperature. Proteins were subsequently precipitated by first adding 16 ml of methanol and mixing, followed by 4 ml of chloroform and mixing. About 12 ml of milliQ water was then added, and the samples were mixed by vortexing. Samples were subsequently centrifuged for 15 min at 11,000*g*, followed by another 5 min at 11,000*g* (this resulted in better separation of the two phases than one round of 20 min of centrifugation). The protein precipitates at the interphase were collected and washed twice with 2 ml methanol, followed by 5 min centrifugation at 11,000*g*. The pellets were then air-dried and resuspended in 5 ml 25 mM NH_4_HCO_3_ (pH 8.4). Trypsin (Worthington) was then added at a ratio of 1:25 (w/w), and overnight digestions at 37 °C were performed.

The next day, samples were centrifuged for 10 min at 11,000*g*, and the supernatants were collected. Desalting of the samples was subsequently performed using HLB Oasis 1 cc cartridges (Waters). Briefly, the cartridge was washed once with 1 ml acetonitrile/water 90/10 (v/v), equilibrated with milliQ water/acetonitrile/formic acid (FA) 95/3/0.1 (v/v/v) (solution A, two times with 1 ml). Following sample loading, the column was washed with solution A (three times with 1 ml), after which peptides were eluted with acetonitrile/water/FA 30/70/0.1 (v/v/v). Tryptic peptides were lyophilized and stored at −20 °C until use.

### Fe^3+^-IMAC

A one-step enrichment procedure was performed using a 4 × 50 mm ProPac IMAC-10 analytical column (Thermo Fisher Scientific), which was stripped using 50 mM EDTA/0.5 M NaCl (pH 4) and charged with 25 mM FeCl_3_ in 100 mM acetic acid, according to the manufacturer's instructions, using an offline pump system (Shimadzu). The column was connected to an Agilent 1200 chromatography system running at a flow rate of 0.3 ml/min. Prior to sample loading, the column was equilibrated in solvent A (water/acetonitrile/trifluoroacetic acid 70/30/0.07 [v/v/v]) for 30 min at a constant flow rate of 0.3 ml/min. Tryptic peptides (10 mg, resuspended in 260 μl solvent A, 250 μl injected) were subsequently loaded, and nonbinding peptides were removed by washing the column for 20 min with solvent A. Peptide separation was then performed using a linear gradient from 0 to 45% of solvent B (0.5% v/v NH_4_OH). The peak fraction of IMAC-bound peptides was manually collected and lyophilized.

### LC–MS/MS analysis

Lyophilized peptides were reconstituted in 100 μl water/FA (100/0.1 [v/v]) and analyzed by on-line C18 nanoHPLC MS/MS with a system consisting of an Easy nLC 1200 gradient HPLC system (Thermo Fisher Scientific) and an Orbitrap Fusion Lumos Tribrid mass spectrometer (Thermo). Samples (10 μl) were injected onto a homemade precolumn (100 μm × 15 mm; Reprosil-Pur C18-AQ 3 μm; Dr Maisch) and eluted on a homemade analytical nano-HPLC column (30 cm × 75 μm; Reprosil-Pur C18-AQ 3 μm). The gradient was run from 2% to 36% solvent B (water/acetonitrile/FA 20/80/0.1 [v/v/v]) in 120 min. The nano-HPLC column was drawn to a tip of ∼5 μm, which acted as the electrospray needle of the MS source. The LUMOS mass spectrometer was operated in data-dependent MS/MS mode for a cycle time of 3 s, with an HCD normalized collision energy of 32% and recording of the MS2 spectrum in the Orbitrap. In the master scan (MS1), the resolution was 120,000, the scan range was *m/z* 300 to 1500, at an automatic gain control target of 400,000 at maximum fill time of 50 ms. Dynamic exclusion was set after n = 1 with exclusion duration of 60 s. Charge states 2 to 4 were included. For MS2, precursors were isolated with the quadrupole with an isolation width of 1.2 Da. First mass was set to 110 Da. The MS2 scan resolution was 30,000 with an automatic gain control target of 50,000 at a maximum fill time of 60 ms.

For additional in-depth analysis of some predefined precursors, EThcD and in addition some MS3 and MS4 experiments were performed. EThcD was performed at standard charge-dependent electron transfer dissociation settings and a supplemental collision activation of 15%. For MS3 and MS4 experiments, selected predefined precursor masses were subjected to MS2 with an HCD collision energy of 32%, and specific predefined fragment ion masses were selected in the ion trap for HCD MS3 with read out in the Orbitrap at a resolution of 60,000. MS4 experiments were performed similarly as MS3, with an additional round of fragment selection in the ion trap followed by read out in the Orbitrap. For the MS analysis of 2-AEP, a solution at a concentration of 100 pmol/μl (in water/acetonitrile/FA [50/50/0.1, v/v/v]) was directly infused into the mass spectrometer at 5 μl/min using a syringe pump. All MS2 and MS3 settings for these infusion experiments were as described for the specifically predefined selected precursor masses.

### Data analysis

MS data were imported in XCalibur Qual Browser (version 2.2; Thermo Fisher Scientific) for manual interpretation and annotation of the spectra. For database searches, XCalibur raw files were converted into peak lists using Protein Discoverer 2.4.0.305 (Thermo Fisher Scientific) and searched against the *C. difficile* strain 630 database (UniProt; 3762 entries, downloaded June 12, 2020) using the Mascot search algorithm (version 2.2.07; Matrix Science). The MS tolerance was set at 10 ppm, and the MS/MS tolerance was set at 20 milli mass units. Trypsin was selected as enzyme (allowing two missed cleavages), and carbamidomethylation was set as fixed modification on cysteine residues. As variable modifications, oxidation (methionine), acetylation (N-terminal protein), and type A (C_13_H_23_O_10_N_2_P, delta mass 398.109 a.m.u.), type A minus methyl (C_12_H_21_O_10_N_2_P, delta mass 384.093 a.m.u.), phosphoHexNAc, and HexNAc (all on Ser and Thr) were selected. A target false discovery rate of 0.01 at the peptide level (based on a target-decoy analysis) was used. As an additional constraint, for peptides modified with a type A structure (or truncations thereof), only peptides with a Mascot ion score above 20 were used.

## Data availability

The MS proteomics data have been deposited to the ProteomeXchange Consortium *via* the PRIDE (39) partner repository with the dataset identifier PXD029552.

## Supporting information

This article contains supporting information.

## Conflict of interest

The authors declare that they have no conflicts of interest with the contents of this article.
