# Supplementary material for: New insights into the type A glycan modification of Clostridioides difficile flagellar protein flagellin C by phosphoproteomics analysis
Source: J Biol Chem. 2022 Jan 20;298(3):101622. doi: 10.1016/j.jbc.2022.101622 (PMC8861647; doi:10.1016/j.jbc.2022.101622)
Supplement: Supplemental Figures S1–S5 [file mmc1.pdf]

Supporting Information

New insights into *C. difficile* strain 630 flagellin C modifications by phosphoproteomics analysis

Paul J. Hensbergen[‡*], Arnoud H. de Ru[‡], Annemieke H. Friggen[†], Jeroen Corver[†], Wiep Klaas Smits[†], Peter A. van Veelen[‡]

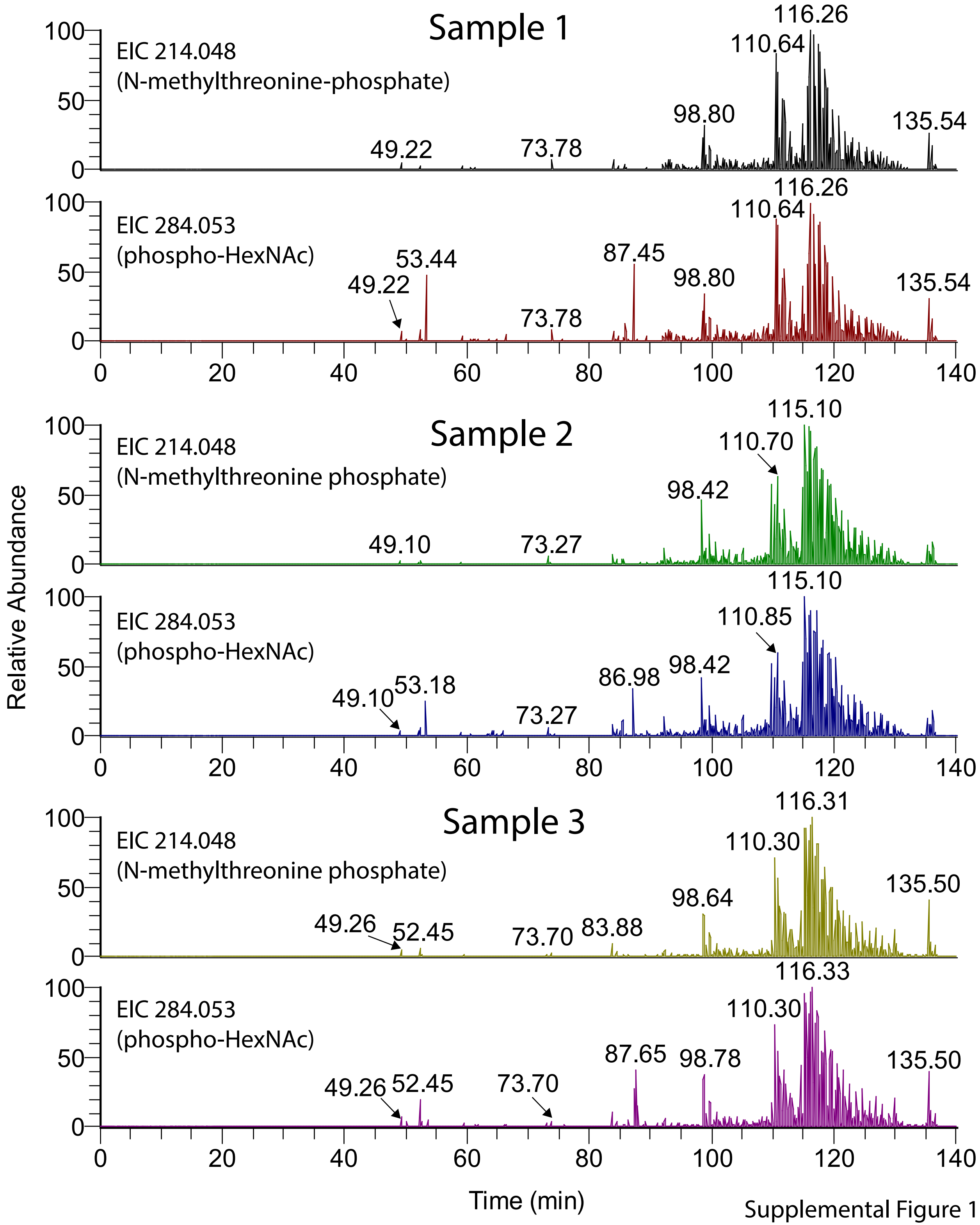

Supplemental Figure 1

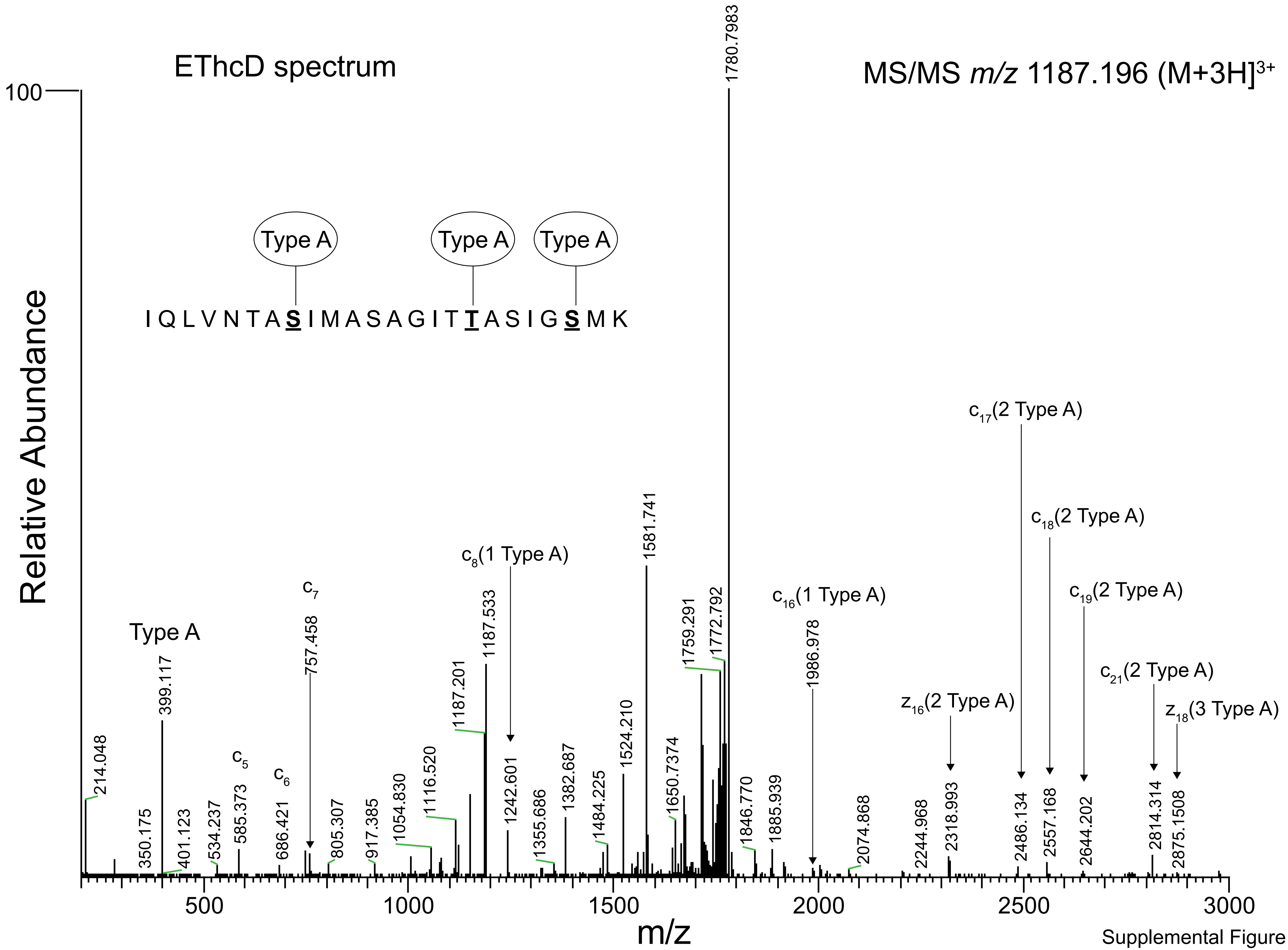

Supplemental Figure 2

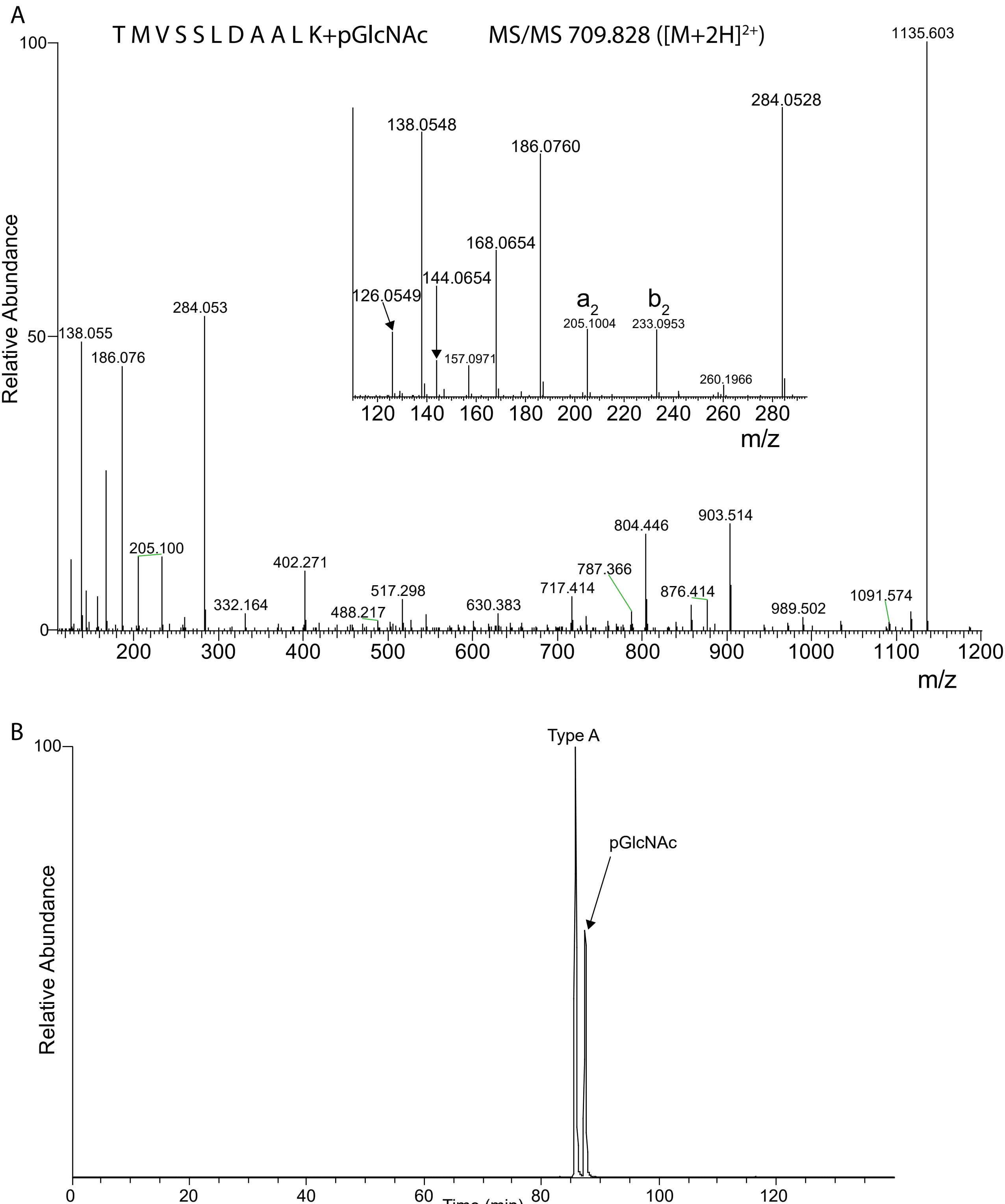

Supplemental Figure 3

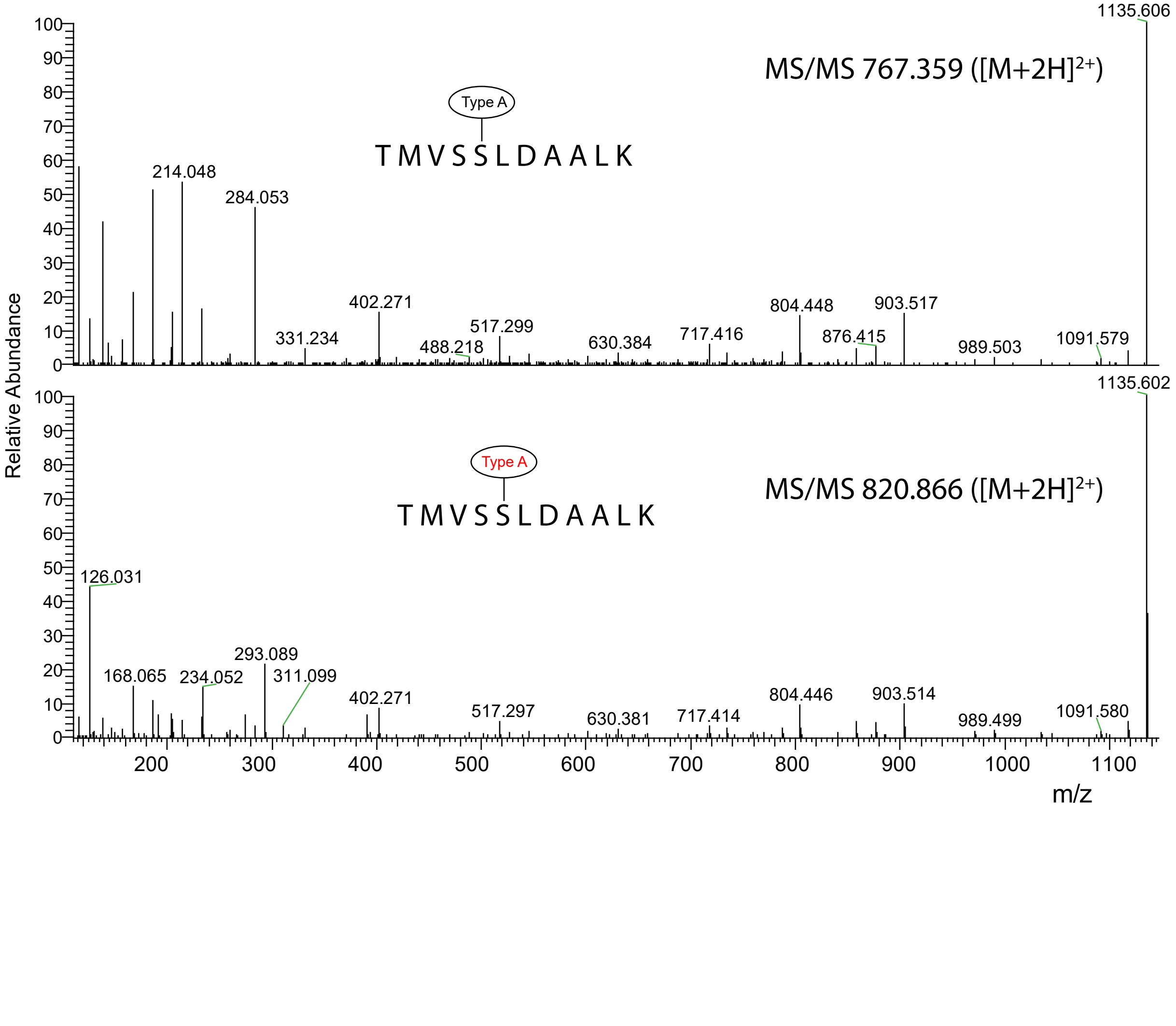

Supplemental Figure 4

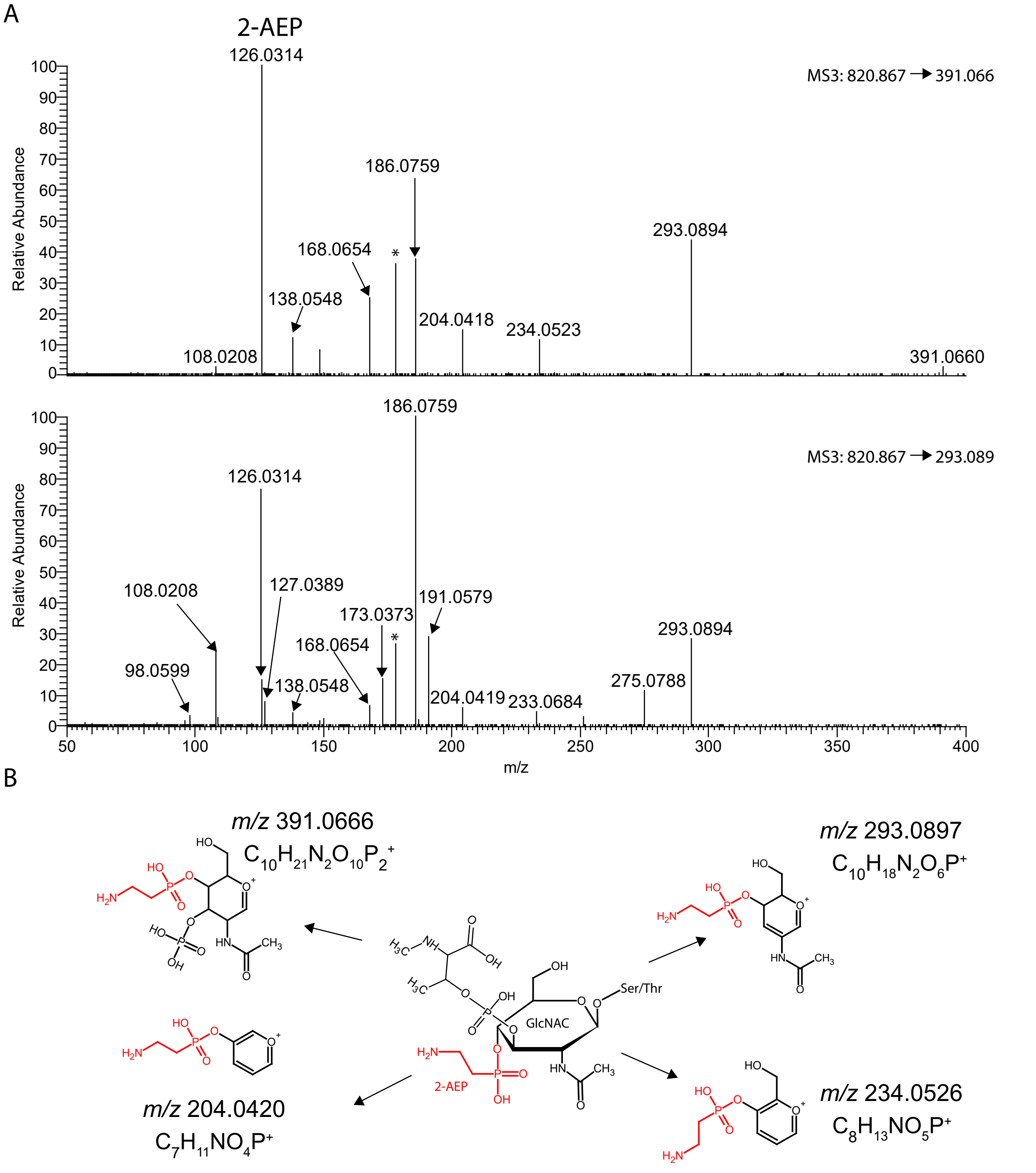

Supplemental Figure 5

Figure legend Supplemental Figures

Supplemental Figure 1. Comparison of extracted ion chromatograms of Type A specific fragment ions in LC-MS/MS data from IMAC purified peptides from three independent *C. difficile* cultures.

Supplemental Figure 2. EThcD of the flagellin C tryptic peptide IQLVNTASIMASAGITTASIGSMK carrying three Type A structures (see Figure 2A).

Supplemental Figure 3. A. MS/MS spectrum of the flagellin C tryptic peptide TMVSSLDAALK carrying a phosho-GlcNAc (pGlcNAc). B. Chromatographic profile of the tryptic peptide TMVSSLDAALK modified with either a Type A structure or a pGlcNAc.

Supplemental Figure 4. Comparison of the MS/MS fragmentation spectrum of the flagellin C tryptic peptide TMVSSLDAALK carrying either a Type A structure (upper panel) or an alternative Type A structure (indicated in red) with an additional mass of 107.013 Da (lower panel, fully annotated in Figure 4). Subsequent analyses showed that this alternative Type A structure corresponds to 2-AEP-Type A (see Figure 4 for further details).

Supplemental Figure 5. A. MS3 fragmentation of the flagellin C tryptic peptide TMVSSLDAALK carrying a 2-AEP-Type A structure, where either the fragment ion at *m/z* 391.066 (upper panel) or 293.089 (lower panel) were selected for additional fragmentation. See Figure 4A for the MS2 fragmentation spectrum. B. Tentative assignment of 2-AEP containing fragments observed in the MS2 and MS3 spectrum of the flagellin C tryptic peptide TMVSSLDAALK carrying a 2-AEP-Type A structure. The signal indicated with an asterisk (*) at *m/z* 178.243 is a background signal of unknown origin which was observed in all fragmentation spectra.
